# Large *de novo* ascending aortic thrombus successfully treated with anticoagulation

**DOI:** 10.15171/jcvtr.2018.18

**Published:** 2018-05-21

**Authors:** Mustafa Houmsse, Asia McDavid, Ahmet Kilic

**Affiliations:** ^1^Metro Early College High School, Columbus, OH 43210, USA; ^2^The Division of Cardiac Surgery, Department of Surgery; The Ohio State University Wexner Medical Center, Columbus, OH 43210, USA; ^3^The Division of Cardiac Surgery, Department of Surgery, The Johns Hopkins Hospital, Baltimore, MD 21287, USA

**Keywords:** Thrombus, Ascending Aorta, Apixaban

## Abstract

An ascending aortic thrombus is a rare source for embolic transient ischemic attack (TIA) or stroke without an associated aortic pathology. Here we describe a case of a patient who presented with generalized symptoms of headache and fatigue who, on subsequent work-up , was found to have an ascending aortic thrombus with no obvious associated aortic pathology, and was successfully treated with apixaban, a newer direct oral anticoagulant.

## Case Report

**Figure 1:**
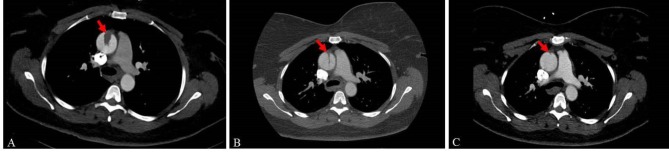
Initial CT at presentation showing ascending aortic thrombus (A), 1-month follow up indicating reduction in size (B), 1-year follow up with further reduction in size (C).

A 49-year-old female presented to the emergency room with headache, fatigue and decreased stamina. Her past medical history was significant for diabetes, hypertension and prior stroke. Her work-up included a trans-thoracic and trans-esophageal echocardiogram, magnetic resonance imaging (MRI), laboratory evaluation, and computed tomography of her aorta (CTA). A mass was found in the ascending aorta, consistent with an aortic mural thrombus, measuring 1.6 × 1.0 × 1.9 cm with some degree of mobility (Figure 1A).

The patient did not have a history of rheumatic disease or underlying bleeding or clotting disorders. Hypercoagulability testing, including antiphospholipid antibody, beta 2 glycoprotein, factor V Leiden, lupus anticoagulant, and prothrombin gene mutation were all negative. After 48 hours of inpatient observation, with resolution of symptoms, the patient requested to be discharged. Following a thorough discussion of the indications, risks and benefits of surgical intervention, the patient opted for trial of medical therapy. She was discharged on anticoagulation with apixaban, chosen for its immediate therapeutic effect given the patient’s wishes to be discharged, with plans for repeat radiographical surveillance and close clinical monitoring, including surgical intervention for symptoms, increase in size of the thrombus, any embolic phenomenon, aortic valvular involvement, or coronary arterial compromise.

The patient was followed for 15 months after her initial presentation with no further symptoms. Serial surveillance CTAs showing resolution of her aortic thrombus are shown (Figure 1B, 1C). There was no discernable associated pathology leading to this thrombus on multiple phases of contrast and the patient was taken off anticoagulation after her latest visit.

## Discussion

An ascending aortic thrombus is a very rare source of embolic TIA or stroke without an associated aortic pathology, such as aortic dissection, atherosclerosis, or aortic aneurysm. There have only been prior case reports in the ascending aorta^1,2^ and descending aorta.^3^ The exact mechanism of thrombus formation in these patients is not well understood.^4^ Aortic surgery or treatment with anticoagulation agents are common management strategies that have been described previously.^4-6^ Here we describe a rare case of *de novo* ascending aortic mural thrombus that may or may not have contributed to the patient’s presentation and describe the first use of the newer direct oral anticoagulants in treating this phenomenon successfully.

Apixaban (ELIQUIS, Bristol-Myers Squibb Company, New York, NY) is a direct oral anticoagulant that works via direct factor Xa inhibition. It is currently Food and Drug Administration approved for non-valvular atrial fibrillation, prevention of venous thromboembolism, treatment and prevention of pulmonary embolisms, and deep vein thrombosis.^7^ We are reporting a unique diagnostic finding and treatment of embolic transient ischemic attack originating from the ascending aortic thrombus without any aortic pathologies (dissection, aneurysm, or traumatic aortic injury). There is no reported literature regarding the utilization of apixaban in treating arterial mural thrombus when it comes to the ascending aorta and we believe this to be the first case report of direct oral anticoagulant efficacy in treating a thrombus in the arterial system.

## Ethical approval

Informed consent was obtained from the Patient for publication of this case report.

## Competing interests

All authors declare no competing financial interests exist.
